# Fractional Anisotropy changes in Parahippocampal Cingulum due to Alzheimer’s Disease

**DOI:** 10.1038/s41598-020-59327-2

**Published:** 2020-02-14

**Authors:** Josué Luiz Dalboni da Rocha, Ivanei Bramati, Gabriel Coutinho, Fernanda Tovar Moll, Ranganatha Sitaram

**Affiliations:** 1 Faculté de psychologie et des sciences de l’éducation, University of Geneva, Geneva, Switzerland; 2 D’Or Institute for Research and Education, Rio de Janeiro, Brazil; 3 Federal Univerisity of Rio de Janeiro, Rio de Janeiro, Brazil; 4 Institute for Biological and Medical Engineering, Department of Psychiatry, and Section of Neuroscience, Pontificia Universidad Católica de Chile, Santiago, Chile

**Keywords:** Diagnostic markers, Alzheimer's disease

## Abstract

Current treatments for Alzheimer’s disease are only symptomatic and limited to reduce the progression rate of the mental deterioration. Mild Cognitive Impairment, a transitional stage in which the patient is not cognitively normal but do not meet the criteria for specific dementia, is associated with high risk for development of Alzheimer’s disease. Thus, non-invasive techniques to predict the individual’s risk to develop Alzheimer’s disease can be very helpful, considering the possibility of early treatment. Diffusion Tensor Imaging, as an indicator of cerebral white matter integrity, may detect and track earlier evidence of white matter abnormalities in patients developing Alzheimer’s disease. Here we performed a voxel-based analysis of fractional anisotropy in three classes of subjects: Alzheimer’s disease patients, Mild Cognitive Impairment patients, and healthy controls. We performed Support Vector Machine classification between the three groups, using Fisher Score feature selection and Leave-one-out cross-validation. Bilateral intersection of hippocampal cingulum and parahippocampal gyrus (referred as parahippocampal cingulum) is the region that best discriminates Alzheimer’s disease fractional anisotropy values, resulting in an accuracy of 93% for discriminating between Alzheimer’s disease and controls, and 90% between Alzheimer’s disease and Mild Cognitive Impairment. These results suggest that pattern classification of Diffusion Tensor Imaging can help diagnosis of Alzheimer’s disease, specially when focusing on the parahippocampal cingulum.

## Introduction

Alzheimer’s disease (AD) is a neurodegenerative disease and the most frequent type of dementia in the elderly. The most common first symptom of AD is a deficit to learn new information. Progression of AD to other brain regions associates with severe cognitive decline that, at a dementia stage, causes disruption of daily routines, personality change, inability to recognize close relatives, loss of communication skills, inability to execute motor tasks, and death. The current treatments of AD are only symptomatic, and none of the treatments is currently able to stop the progression of mental deterioration^1^.

Anatomical, physiological and biochemical biomarkers that reflect specific features of AD have become relevant candidates to be incorporated in the diagnostic criteria^2^. These biomarkers include extracellular deposits of amyloid-ß protein^3^, stages of intraneuronal neurofibrillary tangles of tau protein^4^, and neuritic plaque score^5^. Cerebrospinal fluid (CSF) concentrations of amyloid-ß and tau protein are therefore two potential biomarkers^6^. Preliminary results also suggest amyloid positron emission tomography imaging for clinical diagnosis AD^7^. Moreover, the use of blood-based biomarkers is also a feasible technique. A set of ten lipids from peripheral blood was used as features to predict AD within 2–3 years with over 90% accuracy^8^. Amyloid-ß concentrations in CSF already changes 5–10 years before the onset of clinical AD^9^. Invasive techniques, such as lumbar puncture, have shown efficacy in identifying the individual risk of future development of AD^10^, but the safety of the procedure is controversial^11^.

Non-invasive techniques for detecting AD would be very helpful, considering the possibility of early treatment of prospective patients in the worldwide population. Early diagnosis may relate to better prognostics given that treatment may start in the absence of such significant brain degeneration^12^. In terms of imaging-based diagnosis, hippocampal volumetry has also been proposed as a biomarker for AD^13^, as significant atrophy of the hippocampal formation demonstrated by MRI has identified preclinical stages of AD with 80% accuracy^14^. Two other structural MRI based approaches discriminated AD patients and healthy controls with 88% accuracy in both studies^15,16^. Diffusion Tensor Imaging (DTI) is also a promising imaging technique whose development may provide much earlier evidence of the disease than the neuropsychological symptoms^17^. The Alzheimer’s Disease Neuroimaging Initiative (ADNI) added DTI among several other imaging techniques in an effort to identify reliable biomarkers of AD^18^.

Machine learning approaches for classification between AD and controls based on fractional anisotropy (FA) as input features attained classification accuracies in the range of 75%-88%^16,18–20^. FA decrease in AD patients revealed changes in the parahippocampal white matter^16,19,21^, uncinate fasciculus^16,22,23^, superior longitudinal fasciculus^16,22–24^, cingulum^16,22–24^, fornix^19,22,23^, genu and splenium of corpus callosum^24^. A recent classification based on DTI graph measures^25^ has also achieved 80% accuracy for AD versus healthy controls. A multilevel classification techquine^26^ combining FA values (voxel-level), fiber tracking (connection-level), and graph measures (network-level) achieved 90% accuracy between AD and controls. Multimodal MRI Analysis, combining DTI and fMRI achieved a comprehensive classification accuracy among AD, MCI patients and controls of 92%^27^.

Mild cognitive impairment (MCI) refers to a cognitive decline in absence of dementia. It may indicate a transitional stage between healthy conditions and dementia^28^, including prodromal stages of AD or mild stages of other dementing disorders^29^. The criteria for diagnosis of MCI status include neuropsychological measures, such as Mini-Mental State Examination, Wechsler Adult Intelligence Scale-Revised, Wechsler Memory Scale-Revised, Dementia Rating Scale, Free and Cued Selective Reminding Test, and Auditory Verbal Learning Test^30^. MCI is associated with high risk for the development of AD, with conversion rates between 10% and 15% per year^31^. Therefore, prodromal AD is often categorized as amnestic MCI^29^.

## Methods

Here we propose a FA-based machine learning approach for detecting AD (distinguishing from MCI and healthy controls) focusing on specific areas whose connectivity abnormalities has been frequently reported in literature to be associated with AD: parahippocampal white matter, uncinate fasciculus, superior longitudinal fasciculus, cingulum in the hippocampal formation, cingulum in the cingulate gyrus, fornix, splenium of corpus callosum, and genu of corpus callosum.

### Data acquisition

We recruited 45 elderly adults for DTI data acquisition, including 15 AD patients, 15 MCI patients, and 15 cognitively healthy adults. They were referred for neuropsychological evaluation by their physicians because of memory complaints to discriminate among normal aging, MCI or dementia. Diagnoses were made by a senior board-certified psychiatrist in conjunction with clinical, neuropsychological and MRI assessments collected by a multidisciplinary team of neurologists, neuropsychologists and speech-language therapists. AD diagnoses were performed considering NINCDS-ADRDA criteria^32^. MCI diagnosed patients used in this study were restricted to amnestic subtype, according to the gold standard definitions^33^. Healthy control adults were selected by matching the age and education level to the MCI patients and AD patients, and evaluated considering clinical and cognitive tests (Table 1). This study was approved by the Ethics Committee of D’Or Institute for Research and Education.

**Table 1:** The adults participating in the study.

Subject ID	Controls	MCI	AD	ANOVA (p-value)
Participants	15	15	15	—
Sex	73%F/27%M	67%F/33%M	60%F/40%M	—
Age (years)	74.6 (±6.9)	74.3 (±6.8)	74.5 (±6.5)	0.992
Education (years)	12.0 (±4.1)	11.9 (±5.0)	12.1 (±4.3)	0.993
Mini-Mental State Examination (MMSE)	26.5 (±2.6)	25.9 (±2.5)	21.4 (±4.8)	0.000
Clock drawing test (CDT)	9.8 (±0.6)	8.7 (±2.5)	7.0 (±3.3)	0.010
Digit span forward	4.9 (±1.1)	4.8 (±0.8)	4.4 (±1.0)	0.341
Digit span backward	3.7 (±0.8)	3.4 (±0.7)	2.8 (±1.0)	0.018

For each subject, T1 and DTI images were acquired in the D’or Institute (Rio de Janeiro, Brazil) on a Philips Achieva 3.0 Tesla magnetic resonance scanner, with 8-channel SENSE head coil. T1-weighted structural images of the participants’ brains were acquired by a gradient recalled echo scanning sequence that had the following parameters: repetition time (TR) = 7.16 milliseconds (msec), echo time (TE) = 3.41 msec, flip angle = 8 degrees, acquisition matrix = 480 × 480 with resolution 0.5 mm × 0.5 mm, and 340 sagittal slices with thickness 0.5 mm. Diffusion Tensor Imaging scans were acquired using a spin echo sequence and had the following parameters: TR = 5620 msec, TE = 65 msec, flip angle = 90 degrees, acquisition matrix = 96 × 96 with resolution 2.5 mm × 2.5 mm, and 60 transversal slices with thickness = 2.5 mm. The DTI sequence was composed of 1 B0 image (non-diffusion weighted) and 32 diffusion weighted images (each one with a different gradient direction) with b-value equal to 1000 sec/mm^2^.

### Data processing

Correction for head motion and eddy current artifact of the DTI images were performed using the FSL’s eddy tool^34^. Echo-planar imaging (EPI) induced susceptibility artifacts correction was performed^35^. FA, a scalar value that describes the degree of anisotropy (directionality dependence) of a diffusion process^36^, was computed at voxel resolution. After that, normalization to MNI space was performed on SPM 12^37^. Segmentation into AAL^38^ and JHU-DTI^39^ atlases were performed on DSI Studio platform^40^.

We performed the analysis firstly in the whole brain and next in specific areas whose connectivity abnormalities are frequently reported in literature to be associated with AD: parahippocampal white matter, uncinate fasciculus, superior longitudinal fasciculus, cingulum in the hippocampal formation, cingulum in the cingulate gyrus, fornix, splenium of corpus callosum, and genu of corpus callosum. Parahippocampal white matter was segmented based on AAL atlas, and the other seven brain areas were segmented based on JHU-DTI atlas.

### Leave-one-out cross-validation

FA values were then loaded into MATLAB, and undertook a feature selection procedure based on Fisher Score^41^, before being used as input features for linear Support Vector Machine (SVM) classification^42^, using parameter estimation C equal to 1. Both feature selection and classification were performed under Leave-one-out cross-validation^43^, for binary classification among the three classes (AD patients, MCI patients, and healthy controls).

Leave-one-out cross-validation is a special case of k-fold cross-validation where k (number of folds) is equal the n (number of subjects from each class). Although leave-one-out and 10-fold has demonstrated similar accuracy levels^44^, 10-fold is considered an alternative to minimize the computational expensive cost of leave-one-out in extremely large sample sizes, while leave-one-out is recommended for small sample sizes^45^.

## Results

### Whole brain

**Figure 1:**
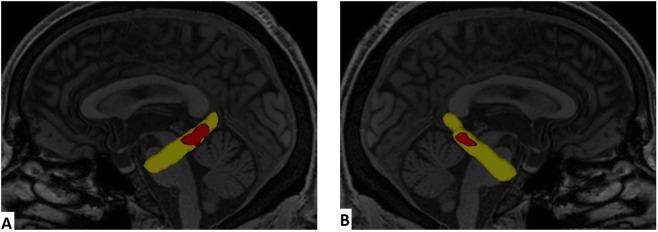
Voxels whose Fisher Score (AD versus healthy controls) are higher than 1 (red) inside bilateral cingulum in hippocampal formation (yellow). **(A)** Left view. **(B)** Right view.

Linear SVM classification (parameter C equal to 1) based on all voxels inside the brain achieved an accuracy of 60% between AD and healthy controls, 57% between AD and MCI patients, and 47% between MCI and controls. Using feature selection, the set of voxels whose Fisher Score were higher than 1.0 reached the highest accuracy between AD patients and healthy controls at 80%, between AD and MCI at 77%, and between MCI and controls at 60%. The two biggest clusters of voxels whose Fisher Scores were higher than 1.0 when comparing AD and controls are inside the bilateral parahippocampal gyrus, as well as inside the bilateral cingulum in hippocampal formation (Fig. 1).

### Analysis in specific brain areas without feature selection

We performed data analysis in specific brain areas: parahippocampal white matter, uncinate fasciculus, superior longitudinal fasciculus, cingulum in the hippocampal formation, cingulum in the cingulate gyrus, fornix, splenium of corpus callosum, and genu of corpus callosum. Therefore, for each analysis, it was included only voxels inside the respective region of interest. Linear SVM classification (parameter C equal to 1) between AD patients and healthy controls without feature selection achieved higher accuracy in both Cingulum in the hippocampal formation or parahippocampal gyrus, among these regions (Table 2). For AD versus MCI patients, cingulum in the hippocampal formation was the brain region with higher discrimination accuracy.

**Table 2:** SVM classification accuracy without feature selection in specific brain areas.

Brain area	AD vs Controls	AD vs MCI	MCI vs Controls
Cingulum in the hippocampal formation	77%	83%	57%
Parahippocampal gyrus	77%	60%	47%
Cingulum in the cingulate gyrus	50%	43%	63%
Genu of the corpus callosum	70%	53%	67%
Splenium of the corpus callosum	63%	43%	47%
Uncinate fasciculus	53%	43%	43%
Fornix	47%	53%	50%
Superior longitudinal fasciculus	57%	47%	50%

### Analysis in specific brain areas with feature selection

As Fisher Score feature selection from all the voxels of the whole brain revealed the two biggest scoring clusters, bilaterally, inside both hippocampal cingulum and parahippocampal gyrus, we performed FA analysis in these bilateral white matter regions, separately.

### Cingulum in the hippocampal formation

**Figure 2:**
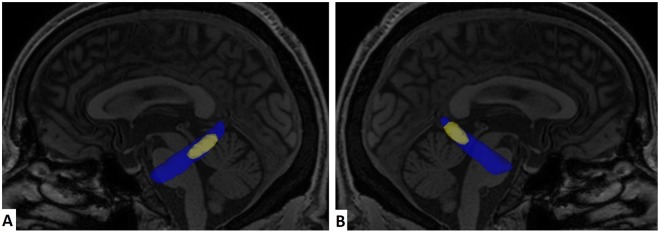
Voxels inside bilateral hippocampal cingulum (blue), and those whose Fisher Score was higher than 0.4 (yellow). **(A)** Left view. **(B)** Right view.

**Figure 3:**
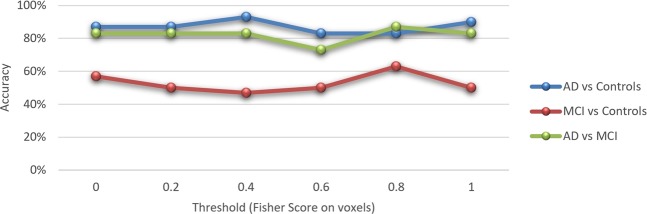
Linear SVM accuracy based on FA for different threshold values of Fisher Score on voxels belonging to bilateral hippocampal cingulum.

Considering only voxels on the hippocampal cingulum whose training samples indicate a decrease in FA values from healthy controls to AD patients, SVM achieved average accuracies of 87% between AD and controls, 83% between AD and MCI, and 57% between MCI and controls. Considering only voxels whose training samples indicate an increase on FA from healthy controls to AD patients, SVM achieved accuracies of 47% for all three binary permutations of subject groups. Therefore, the discriminative voxels in the bilateral hippocampal cingulum show a decrease in FA values in AD patients. Applying feature selection on the voxels inside the bilateral hippocampal cingulum, the set of voxels whose Fisher Score was higher than 0.4 (Fig. 2) reached the highest accuracy at 93% between AD and controls. The set of voxels whose Fisher Score was higher than 0.8 attained an accuracy of 87% between AD and MCI, and 63% between MCI and controls (Fig. 3).

#### Parahippocampal gyrus

Considering only voxels on the parahippocampal gyrus whose training samples indicates a decrease in FA values from healthy controls to AD patients, linear SVM reached an accuracy of 83% between AD and controls, 67% between AD and MCI, and 47% between MCI and controls. Considering only voxels whose training samples indicate an increase in FA from healthy controls to AD patients, linear SVM achieved an accuracy of 50% between AD and MCI, and 47% for both AD versus controls and MCI versus controls. These voxels in the bilateral parahippocampal gyrus also reveal a decrease in FA values in AD patients. When feature selection is applied on voxels inside bilateral parahippocampal gyrus, the set of voxels whose Fisher Score were higher than 0.8 reached the highest accuracy at 90% between AD and controls. The set of voxels with Fisher Score higher than 1.2 reached an accuracy of 90% between AD and MCI.

## Discussion

Whole brain Fisher Score feature selection reached up to 80% accuracy, and most of the selected voxels were contained inside bilateral hippocampal cingulum and parahippocampal gyrus. In the bilateral hippocampal cingulum, our classification method achieved the highest accuracy of 93% between AD patients and healthy controls, and 87% betwenn AD and MCI. In the bilateral parahippocampal gyrus, our approach obtained the highest accuracy at 90% in both discriminations involving AD patients: AD versus controls, and AD versus MCI. The features from the voxels selected from the parahippocampal gyrus and the hippocampal cingulum resulted in classification accuracy much higher than in other analyzed brain areas, including cingulum in the cingulate gyrus, genu and splenium of corpus callosum, fornix, uncinate fasciculus and superior longitudinal fasciculus. Based on these results, we can suggest that a stage of specific FA alterations inside the hippocampal cingulum and the parahippocampal gyrus is a potential biomarker for AD.

Our findings in parahippocampal gyrus are in accordance with indications from previous studies that there are FA alterations in the parahippocampal white matter in different stages of AD^19,21^. Parahippocampal gyrus has been implicated in episodic autobiographical memory^46^, whose abnormalities are related to the first AD symptoms, such as a deficit to learn new information. Parahippocampal gyrus is a part of the hippocampal formation. The most important role of the hippocampal formation is in learning and memory functions^47^. FA decline in the hippocampal white matter has also been implicated AD^48^. Hippocampal cingulum is the hippocampal formation’s portion of the cingulum^39^, located inferior to the axial level of the splenium of corpus callosum. The cingulum is a major pathway of the limbic system, connecting the cingulate gyrus to the hippocampal formation^49^. Cingulum contains fibers with different lengths. The longest one connects amygdala, uncus, parahippocampal gyrus and subgenual areas of the frontal lobe^50^. The cingulum is connected to its adjacent areas by perpendicular crossing shorter fibers^51^. The correlation between the appearance of AD symptons and the degeneration of specific long main or short adjacent white matter fibers of the cingulum is not clear yet. Our findings in hippocampal cingulum are also in accordance with most of the previous studies, including connectivity loss in the cingulum bundle in different stages of AD^16,22–24^, and stages of MCI leading to AD^23,24,52^. The subregion at the intersection of hippocampal cingulum (from JHU-DTI atlas) and parahippocampal gyrus (from AAL atlas) can be named parahippocampal cingulum, as proposed in a recent study^53^. Our method and results may help the development of new techniques to diagnose AD based on this abnormality localized in the bilateral parahippocampal cingulum.

In order to avoid a frequent misinterpretation, it is important to point out the differences between cingulum (white matter) bundle and cingulate (gray matter) cortex. While the cingulate cortex covers the cingulum bundle on the frontal and parietal lobes, it does not cover cingulum in the temporal lobe. Vogt *et al*.^54^ suggested that the cingulate cortex could be subdivided into the anterior, mid, posterior and retrosplenial cortices. In the JHU-DTI atlas, the cingulum is separated at the axial level of the splenium of the corpus callosum into the cingulum of the cingulate gyrus and hippocampal cingulum^39^. The cingulum of the cingulate gyrus runs inside the frontal and parietal lobes, while the hippocampal cingulum runs inside the temporal lobe. Recently, Jones *et al*.^53^ propose dividing the cingulum bundle into three subdivisions corresponding to the parahippocampal, retrosplenial, and subgenual portions. An even more recent study has proposed to segment cingulum bundle (CB) into 5 subcomponents: “CB-I runs from the subrostral areas to the precuneus and splenium, encircling the corpus callosum (CC). CB-II arches around the splenium and extended anteriorly above the cingulate cortex to the medial aspect of the superior frontal gyrus. CB-III connects the superior parietal lobule and precuneus with the medial aspect of the superior frontal gyrus. CB-IV is a relatively minor subcomponent from the superior parietal lobule and precuneus to the frontal region. CB-V, the para-hippocampal cingulum, stems from the medial temporal lobe and fans out to the occipital lobes”^55^. In this way, parahippocampal cingulum has been recently understood as a possible location for the earliest exhibition of neuronal degeneration due to AD^55^. Wisse *et al*.^56^ have observed a slight decrease in FA mean of the whole parahippocampal cingulum in AD patients when compared to healthy controls. On the other hand, our approach includes a voxel-wise analysis, with univariate feature (voxel) selection (Fisher score), pattern classification with SVM and leave one out cross-validation. We also have mapped the subregions inside the parahippocampal cingulum where this FA decrease pattern in AD was identified.

MCI is a heterogeneous condition, which includes impairments from a wide spectrum of cognitive functions. Those impairments might be derived from early stages of a wide spectrum of dementias (including AD) or even by non-pathologic causes^57^. Since discrimination between MCI patients and healthy adults based on FA values at voxel resolution inside the hippocampal cingulum and the parahippocampal gyrus were around chance-level, no consistent FA alteration on amnestic MCI in comparison to healthy controls was observed in the above regions. Our analyses indicate that amnestic MCI patients (which theoretically includes patients who are progressing towards AD) do not have substantial FA alterations in the parahippocampal cingulum bundle. However, our approach is currently unable to detect neural integrity abnormalities at a much greater spatial resolution than the millimetric scale. For this reason, we indicate that a substantial decrease in FA values in hippocampal cingulum and parahippocampal gyrus occurs only in AD patients, but not in amnestic MCI patients. On the other hand, a progressive FA decrease from health through different stages of MCI and AD has also been reported^23^. However, our present study did not systematically record the mean delayed recall scores for detecting memory impairment levels on amnestic MCI subjects. Therefore, due to this limitation, we suggest further investigation on amnestic MCI levels of axonal integrity in the bilateral parahippocampal cingulum bundles, considering only MCI subjects having confirmed AD pathology (amyloid-positive marker).

Therefore, the identification of white matter connectivity damage levels in the bilateral parahippocampal cingulum bundle needs to be further investigated in future studies. We also recommend for a future study the consideration of a longitudinal approach including different stages of AD for a better understanding of the progression of the disease from the earliest to the most advanced stages, what has not been considered in our present approach. A better understanding about the progression of neuronal deterioration and its correlation with psychological symptoms may serve as reference for the development of new treatments, which may include real-time neurofeedback^58–60^ and brain-computer interface training^61,62^. The development of new approaches to find biomarkers for predicting the individual risk factors of contracting AD dementia can also be a relevant improvement.

The number of individuals used in the analysis (15 AD patients, 15 MCI and 15 healthy individuals) is a limitation of this study. Although a number of previous works in pattern classification as applied to medical imaging data using support vector machines and leave-one-out cross-validation have shown the accuracy of these novel techniques and their applicability to small sample sizes^63–67^, a study with a more comprehensive population size will be more representative for the worldwide population. This development in imaging and its analysis is motivated by the need to provide data-driven approaches for diagnosis and scientific studies with the practical and cost considerations of small sample sizes. These developments are important for the scientific understanding of the disease as well as clinical diagnosis.

DTI, as an MRI technology, has the advantage of being a non-invasive technique when compared with molecular biomarkers, such as CFS based biomarkers. Results achieved using DTI for AD diagnosis are still less robust than ones achieved using blood-based biomarkers^8^. However, DTI has the capability to evaluate the neuroanatomic evolution of the AD in individual patients, which is not in the scope of blood-based approaches.

The major contributions of this imaging study is the achievement of high classification among Alzheimer’s, MCI and healthy individuals, and also the machine learning based statistical mapping of the brain region (parahippocampal cingulum) directly involved in the diseases, so that it may serve as a potential biomarker of AD for assisting in diagnosis. Furthermore, fractional anisotropy measurements on parahippocampal cingulum can potentially serve in evaluating the progression of neuronal deterioration (or even possible recovering) of AD patients undergoing treatment.

## Conclusion

Our results indicate that DTI approaches are potentially good diagnostic tools for clinical evaluation of AD patients. Based on our results in this AD study, the brain regions indicated to be mainly discriminative between these classes are hippocampal cingulum and parahippocampal gyrus. Whether future approaches based on those features could achieve close to 100% accuracy and be reliable (alone or in combination with other methods) for clinical diagnosis is an important question to be answered. Also for future research, we also would like to remark the importance of the development of new longitudinal studies in order to better understand the evolution of parahippocampal cingulum white matter alterations during the progression of AD and its potential use as a specific biomarker of AD white matter alterations.

## Ethics Approval and Consent to Participate

The imaging acquisition was performed at D’Or Institute for Research and Education. The study was approved by the Ethics Committee of D’Or Institute for Research and Education (Rio de Janeiro, Brazil), as a retrospective survey of clinical files. Patients did not sign informed consent, in accordance with the Brazilian regulations. All procedures involving human participants were in accordance with the 1964 Helsinki declaration.
